# Workplace sustainability: energy-saving behaviors in office environments of Thailand

**DOI:** 10.3389/fpsyg.2025.1400410

**Published:** 2025-03-04

**Authors:** Su Wutyi Hnin, Amna Javed, Jessada Karnjana, Chawalit Jeenanunta, Youji Kohda

**Affiliations:** ^1^School of Management Technology, Sirindhorn International Institute of Technology, Thammasat University, Pathum Thani, Thailand; ^2^School of Knowledge Science, Japan Advanced Institute of Science and Technology, Nomi, Japan; ^3^Intelligent System Research Group, NECTEC, National Science and Technology Development Agency, Pathum Thani, Thailand

**Keywords:** energy-saving behavior, ability–motivation–opportunity model, norm activation model, theory of planned behavior, structural equation modeling

## Abstract

**Introduction:**

Energy consumption in office environments significantly impacts global energy usage, particularly due to lighting, air conditioning, and electronic devices. Urbanization and economic growth in Thailand exacerbate energy demands, positioning office environments as essential for energy conservation efforts. Traditional strategies have primarily focused on technological solutions, but these approaches often fail to address the pivotal role of human behavior in shaping energy consumption.

**Methods:**

This study develops a culturally contextualized framework by integrating the Motivation-Opportunity-Ability (MOA) model, the Norm Activation Model (NAM), and the Theory of Planned Behavior (TPB) to examine key determinants of workplace energy-saving behavior. Data were collected from 105 office workers in Bangkok, Thailand, through an online survey. Using Partial Least Squares Structural Equation Modeling (PLS-SEM), this study validated the framework to analyze the relationships between motivation, opportunity, ability, intention, and behavior within hierarchical workplace structures and collective decision-making settings.

**Results:**

The results highlight motivation and ability as significant predictors of energy-saving behavior, reinforcing the role of awareness of consequences, personal norms, and perceived control. However, opportunity, intention, and individual comfort exhibit negative relationships with energy-saving behavior, suggesting that structural policies, behavioral intentions, and thermal satisfaction interact in complex ways.

**Conclusion:**

These findings underscore the importance of contextually adaptive workplace policies that account for behavioral and structural energy conservation challenges. By providing a culturally sensitive framework, this study offers insights for policymakers and organizational leaders to develop effective and sustainable energy-saving strategies that integrate behavioral considerations alongside technological interventions.

## 1 Introduction

Energy consumption in office environments constitutes significant global energy use, driven by lighting, air conditioning, and electronic devices. As hubs of economic and professional activity, office buildings present substantial energy conservation opportunities (Xu et al., 2020; Mantesi et al., 2022). In Thailand, where rapid urbanization and economic growth have intensified energy demands, office environments play a key role in achieving national energy efficiency goals. Efforts to reduce energy consumption in offices have primarily focused on technological solutions, such as energy-efficient lighting and automated HVAC systems, which have shown measurable success. However, these methods often overlook the critical role of human behavior in shaping energy consumption patterns. Behavioral interventions, such as awareness campaigns and reminders, have also been employed but frequently lack the contextual relevance and specificity needed to influence sustained energy-saving practices, particularly in diverse and culturally nuanced settings like Thailand (Su et al., 2022; Akhound et al., 2022). Recent research has provided valuable insights but reveals significant gaps in understanding energy-saving behaviors in office environments. Akhound et al. (2022) explored employees' intentions to reduce workplace energy consumption, highlighting the influence of employee attitudes and perceived organizational support. However, their findings lacked integration with theoretical models that comprehensively explain behavioral drivers. Similarly, Su et al. (2022) examined the role of organizational energy-saving culture using system dynamics modeling, emphasizing the importance of fostering an energy-conscious culture while neglecting motivational and psychological factors. Xu et al. (2020) studied the impact of social norms. They ascribed responsibility for energy-saving behaviors across different office layouts, identifying behavioral variations linked to workplace design but failing to integrate these findings with broader behavioral theories, such as the Theory of Planned Behavior (TPB). Mantesi et al. (2022) analyzed post-pandemic energy consumption trends in office environments, emphasizing the importance of adaptive energy management strategies but noting the lack of focus on individual employee behaviors. These studies collectively highlight the need for a comprehensive framework that integrates individual, social, and organizational factors to address the unique challenges of energy conservation in office environments, particularly in Thailand. This research addresses these gaps by integrating the Motivation-Opportunity-Ability (MOA) framework with constructs from the Norm Activation Model (NAM) and TPB to develop a holistic model for understanding energy-saving behaviors in office environments. This study uses Partial Least Squares Structural Equation Modeling (PLS-SEM) to analyze survey data collected from office workers in Thailand. The proposed framework examines how ability, motivation, opportunity, and behavioral intentions interact while incorporating external organizational influences such as workplace culture and social norms. By focusing on office environments in Thailand, the research provides culturally contextualized insights into energy-saving practices, extending the applicability of global behavioral theories to regional and organizational contexts. The contributions of this research are four-fold. Theoretically, it advances workplace sustainability studies by integrating three well-established behavioral models—MOA, NAM, and TPB—into a unified framework. Methodologically, it demonstrates the utility of PLS-SEM in analyzing complex relationships between individual and organizational factors in office settings. Practically, it provides actionable recommendations for policymakers and organizational leaders to design effective energy-saving interventions tailored to office workers' motivations and workplace dynamics. Contextually, it bridges global theories with Thailand's unique cultural and managerial practices, offering a framework to guide energy conservation strategies in diverse office environments. These contributions aim to promote more effective and sustainable energy-saving practices in Thailand and beyond. The remainder of the article is structured as follows: Section “Related works” defines the research framework and hypotheses, focusing on energy-saving behaviors in office workplaces. Section “Research framework and hypothesis” presents the research methods and data collection process. Section “Analysis of data and results” analyzes the data and results, while Section “Discussion and implications” discusses the findings and implications. Finally, Section “Conclusion” concludes the study and outlines potential directions for future research.

## 2 Related works

### 2.1 Energy consumption in office environments

Energy consumption in office environments accounts for a substantial portion of global energy use, primarily driven by the demand for lighting, air conditioning, and electronic devices. Office buildings, as centers of economic and professional activity, offer significant opportunities for energy conservation. Technological interventions such as Personalized Environmental Control Systems (PECS) and advanced dimming systems have shown promise in optimizing energy efficiency. Bian and Hu (2024) conducted a study that utilized neural networks to optimize dimming technologies, achieving a balance between visual comfort and energy efficiency. Organizational strategies also play a vital role in conservation efforts. Su et al. (2022) emphasized the importance of fostering an energy-conscious workplace culture, which can significantly reduce consumption through behavioral change. As Mantesi et al. (2022) examined, post-pandemic hybrid work models further underscore adaptive energy management's importance in accommodating shifting office usage patterns. Behavioral aspects remain critical in achieving energy efficiency. Li et al. (2019) applied the Motivation-Opportunity-Ability (MOA) framework, demonstrating how individual and organizational factors shape energy-saving behaviors. Weerasinghe et al. (2023) highlighted the social psychological dynamics influencing occupant energy-related behaviors, particularly the interplay of peer influence and managerial support in fostering sustainable practices.

### 2.2 Behavioral frameworks in energy conservation

Behavioral theories such as the MOA framework, Norm Activation Model (NAM), and Theory of Planned Behavior (TPB) have been extensively used to explain energy-saving behaviors in office environments. The MOA framework emphasizes motivation, opportunity, and ability as key action enablers. Li et al. (2019) demonstrated its effectiveness in fostering energy-saving behaviors by linking employee motivation with organizational enablers. However, it underrepresents social and normative influences such as moral responsibility and peer accountability (Zhang et al., 2024). NAM offers a complementary perspective by focusing on personal norms and moral responsibility as drivers of pro-environmental behavior. Tverskoi et al. (2021) found that moral responsibility and social norms significantly impact employees' willingness to adopt energy-saving behaviors. However, NAM's limited consideration of external organizational enablers restricts its applicability to complex workplace settings (Weerasinghe et al., 2023). The TPB bridges some of these gaps by incorporating attitudes, subjective norms, and perceived behavioral control. Xu et al. (2020) applied TPB to understand energy-saving intentions in office layouts, demonstrating the role of perceived control and peer norms in shaping workplace behaviors. Integrated frameworks that combine elements of these models have shown promise. Li et al. (2019) proposed a unified framework integrating MOA's actionable enablers, NAM's moral dimensions, and TPB's emphasis on social influences. Zhang et al. (2024) supported this approach, illustrating how integrated models enhance the understanding of both individual and collective drivers of energy-saving behaviors. From a practical standpoint, behavioral interventions rooted in psychological theories have succeeded (Shrestha et al., 2021). Kotsopoulos et al. (2018) showed that tailored gamification strategies aligned with workplace dynamics significantly engage employees in energy-saving actions. Similarly, Carrus et al. (2021) emphasized psychological predictors, such as intentions and awareness, as critical to designing interventions that promote sustainable practices.

### 2.3 Cultural and regional contexts: focus on Thailand

Cultural and regional dynamics significantly influence energy-saving behaviors, particularly in Thailand, where socioeconomic structures intersect with cultural norms. Donovan et al. (2016) linked higher education and environmental awareness to the increased adoption of sustainable practices in Thailand. Hnin et al. (2024) highlighted the dominance of cost-saving motivations over ecological concerns. These findings emphasize the need for culturally tailored interventions that address practical and environmental motivations. Office environments in Thailand present unique behavioral dynamics. Apipuchayakul and Vassanadumrongdee (2020) demonstrated that cultural attitudes and perceived behavioral control strongly influence the adoption of energy-efficient practices. Similarly, Jareemit and Limmeechokchai (2019) identified socio-economic factors, such as education and income, as critical determinants of energy-saving behaviors in Bangkok households. Environmental factors further shape adaptive behaviors. Iamtrakul et al. (2020) highlighted that urban heat islands in Thai cities encourage energy-saving practices such as reduced air conditioning use.

### 2.4 Identified gaps and modified integrated framework

Despite advancements in understanding energy-saving behaviors, significant gaps persist, particularly in integrating psychological, organizational, and cultural dimensions into comprehensive theoretical models. Frameworks such as the MOA model, NAM, and TPB provide valuable insights but exhibit limitations when applied in isolation. For instance, MOA highlights external enablers and individual abilities but neglects moral and normative influences critical for sustained behavior change (Li et al., 2019). Similarly, NAM emphasizes personal moral responsibility and social norms yet overlooks external organizational enablers and structural opportunities essential in workplace settings (Zhang et al., 2024). TPB extends these models by incorporating subjective norms and perceived behavioral control but lacks attention to cultural and contextual factors that shape behaviors in specific regions, such as Thailand (Xu et al., 2020; Hnin et al., 2024). Thai workplaces present unique challenges due to hierarchical structures, collective decision-making, and cost-saving priorities, significantly influencing energy-saving behaviors (Apipuchayakul and Vassanadumrongdee, 2020; Hnin et al., 2024). Additionally, environmental factors, such as urban heat islands, remain underexplored in existing frameworks but play a critical role in shaping energy use decisions in Thailand's urban office environments (Iamtrakul et al., 2020). These gaps highlight the need for a more contextually sensitive and comprehensive framework. This study proposes a modified integrated framework that builds on MOA, NAM, and TPB while introducing novel elements to address these limitations. The framework incorporates cultural moderators, such as hierarchical workplace structures, to reflect regional dynamics' influence better. It also integrates organizational factors, such as peer influence and energy-saving culture, alongside external environmental considerations like hybrid work arrangements and thermal comfort. By capturing these interactions, the modified framework bridges gaps in the literature and offers actionable insights for designing tailored energy-saving interventions in Thai office environments.

## 3 Research framework and hypothesis

### 3.1 Research framework

**Figure 1:**
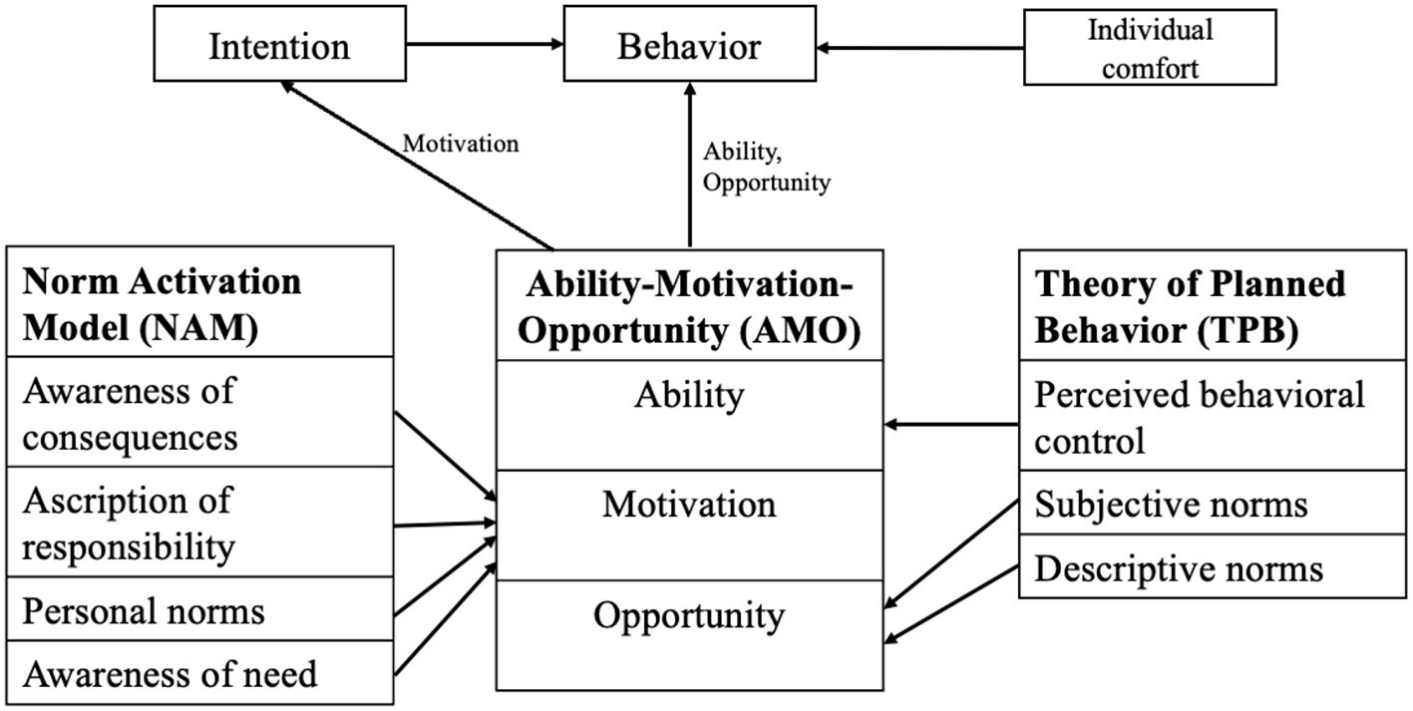
Integration of AMO, NAM, and TPB models.

**Figure 2:**
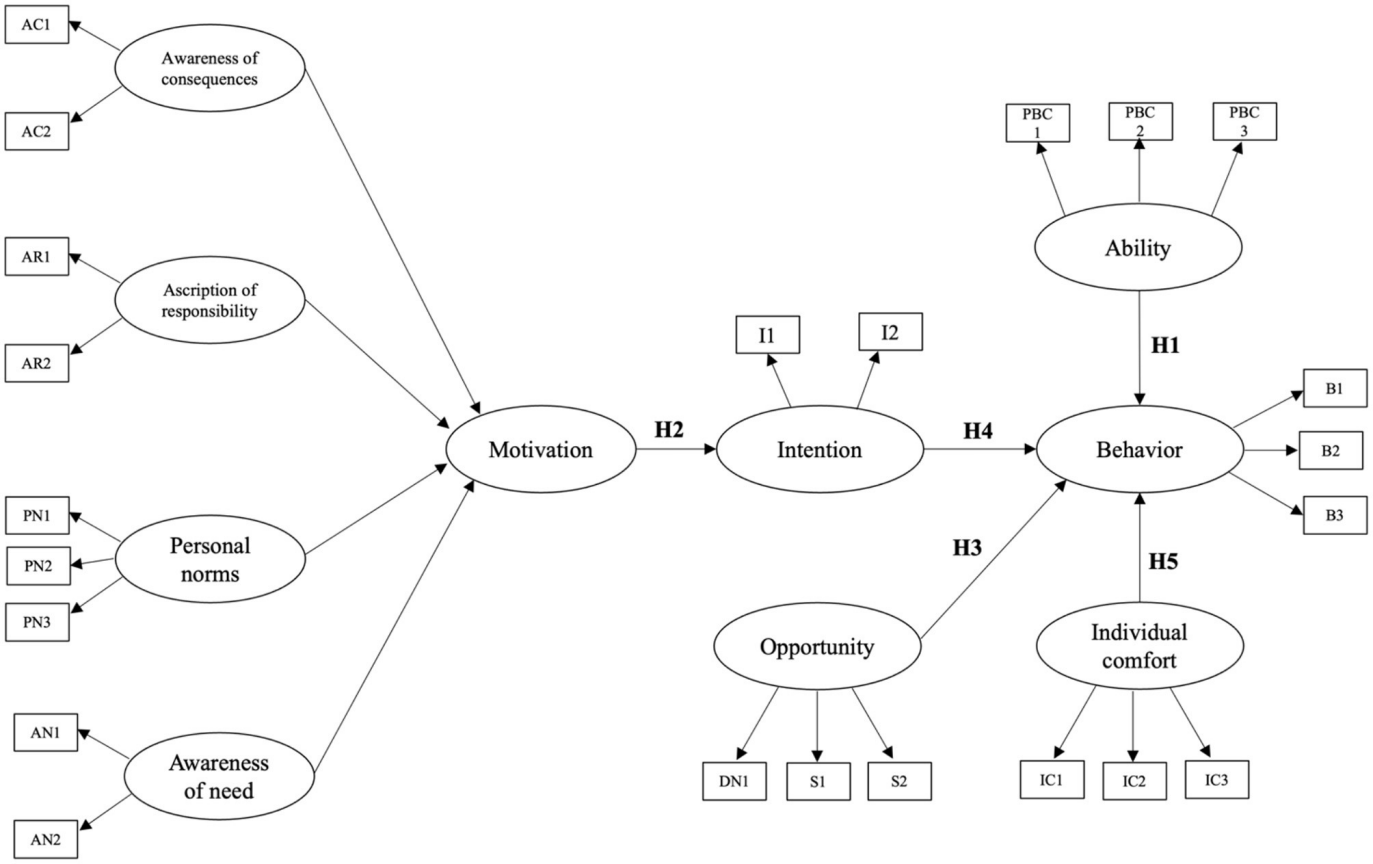
Research framework of private companies' energy-saving behavior in Thailand.

The study employs a modified integrated framework synthesizing the MOA, NAM, and TPB models to understand energy-saving behaviors in office environments comprehensively. The MOA model emphasizes the interplay of motivation, opportunity, and ability, highlighting the role of external enablers and individual capabilities in influencing workplace behaviors. While MOA captures structural and organizational factors, it lacks consideration of moral and normative dimensions, which the NAM addresses. NAM explains pro-environmental behavior through constructs such as awareness of need, awareness of consequences, ascription of responsibility, and personal norms, making it particularly relevant in contexts where moral responsibility drives action. However, NAM does not consider external and organizational factors crucial in workplace settings. TPB complements these models by integrating subjective norms, descriptive norms, and perceived behavioral control to predict intentions and behaviors, bridging the gap between individual decision-making and organizational dynamics. Despite its utility, TPB often underrepresents intrinsic motivations and cultural variations. The proposed framework addresses the limitations of existing models by building upon the Theory of Planned Behavior (TPB) and incorporating cultural moderators, such as hierarchical workplace structures and collective decision-making processes, alongside contextual factors like thermal comfort and hybrid work arrangements. Although TPB integrates subjective norms, descriptive norms, and perceived behavioral control to predict intentions and behaviors, it often underrepresents intrinsic motivations and cultural variations. By including these additional dimensions, the framework effectively captures Thailand's unique environmental and organizational nuances, providing a more comprehensive approach to understanding energy-saving behaviors. The integrated framework comprises five key constructs: Motivation, Opportunity, Ability, Intention, and Individual Comfort. Motivation encapsulates intrinsic psychological drivers, including personal norms, awareness of need, ascription of responsibility, and awareness of consequences, which collectively shape behavioral intention. Intention functions as a mediating mechanism, converting motivational forces into tangible energy-saving behaviors. Opportunity, conceptualized through subjective and descriptive norms, moderates the motivation-intention relationship by incorporating external social and organizational influences. Ability, defined through the construct of perceived behavioral control, delineates an individual's capacity to execute energy-saving actions, exerting a direct influence on behavior while reinforcing the impact of intention. Individual comfort serves as a critical contextual moderator, particularly within Thailand's hot and humid subtropical climate, where energy conservation efforts—such as minimizing air conditioning usage—may conflict with workplace thermal satisfaction. The perceived behavioral control component within the TPB framework further refines the conceptualization of Ability, integrating both physical capacity and the perceived feasibility of performing energy-efficient behaviors. Workplace energy conservation is often contingent on practical considerations; office workers may exhibit reluctance to engage in such practices if they perceive them as burdensome or disruptive to their workflow. By synthesizing these constructs, the proposed framework provides a holistic and contextually adaptive model for understanding energy-saving behaviors in office environments. Figure 1 visually delineates the interrelationships among these constructs, demonstrating the integration of the MOA, NAM, and TPB models into a unified theoretical structure that accounts for individual, social, and environmental determinants of workplace energy conservation. The integrated research framework of the private companies in Thailand is shown in Figure 2.

### 3.2 Research hypothesis

#### 3.2.1 Ability

Ability is the competency to perform a particular mental or physical act or an existing skill and the basic and psychological physical abilities to achieve an outcome (Shi et al., 2017). The PBC of the TPB is used to represent ability as a measurable (Fornara et al., 2016) and indicates the difficulty or ability to perform a single behavior (Fornara et al., 2016; Wang et al., 2018) has made a significant contribution to raising awareness about energy-saving. Workers may be reluctant to save energy in the workplace when the behavior requires physical effort.

Hypothesis 1. Ability has a direct and positive impact on energy-saving behavior.

#### 3.2.2 Motivation

Motivation is usually considered a force that steers individuals toward goals in desired behaviors (Michie et al., 2011). It captures workers' values, needs, participation, and preoccupation in the workplace to represent behavior. Three of the constructs from the NAM are used as social-psychological factors in motivation: personal norms, awareness of consequences, and ascription of responsibility (Kim et al., 2018). Personal norms act as a way of self-testing and an intake requirement to engage in pro-environmental or pro-social behavior. Awareness of need highlights the importance of recognizing the necessity or requirement for a particular behavior, which influences decision-making (Deci and Ryan, 2000). Awareness of consequence features the main construct, expectancy, that affirms the behavior of expectancy drives, which plays an important role in the theory of motivation based on cognition. Ascription of responsibility also plays an important role in motivation theory as a cognitive component (Lopes et al., 2019).

Hypothesis 2. Motivation has a direct and positive impact on intention.

#### 3.2.3 Opportunity

Opportunity refers to an external factor that either inhibits or activates behavior. It integrates interpersonal and environmental factors that limit or facilitate energy-saving behaviors at work. Subjective norms of the TPB are used to treat opportunity as a measurable component. Subjective norms are whether most people disagree or agree with energy-saving behavior. Descriptive norms have also been included as an additional construct in TPB (Forward, 2009). Descriptive norms determine one's beliefs about the behavior of others (Conner et al., 2003).

Hypothesis 3. Opportunity has a direct and positive impact on energy-saving behavior.

#### 3.2.4 Intention

Intention is a fundamental determinant of behavior, representing the level of eagerness individuals possess in making decisions to act. In workplace energy conservation, intention signifies individuals' readiness to turn off unused electronic devices, reduce energy consumption, or adopt energy-efficient practices. It is generally assumed that individuals with strong intentions are more likely to translate their readiness into actual energy-saving behaviors, provided that enabling conditions align with their goals. Understanding intention is critical as it bridges motivational and contextual factors, offering insights into how cognitive and environmental influences shape workplace energy-saving practices (Chai and Baudelaire, 2015).

Hypothesis 4. Intention has a direct and positive impact on energy-saving behavior.

#### 3.2.5 Individual comfort

Saving energy to the detriment of an individual's comfort is not ideal. Individual comfort and energy savings are closely tied together. Setting an energy-saving approach into action at the cost of an individual's comfort was difficult, depending on the discussion of the individual concept of the trade-off between energy-saving and thermal comfort (Li et al., 2019).

Hypothesis 5. Individual comfort has a direct and positive impact on energy-saving behavior.

### 3.3 Summary of hypotheses

The hypotheses proposed in this study provide a structured basis for examining the interplay of psychological, organizational, and contextual factors influencing energy-saving behaviors within workplace settings. The first hypothesis posits that ability, defined as the availability of physical and psychological resources, directly and positively affects energy-saving behavior. Equipping individuals with the necessary skills and tools enables effective energy conservation efforts. The second hypothesis emphasizes motivation as a key driver of intention, incorporating intrinsic factors such as personal norms, moral responsibility, and awareness of consequences, which collectively inspire proactive energy-saving decisions. Opportunity, as conceptualized in the third hypothesis, refers to external enablers such as supportive policies and organizational structures posited to facilitate energy-saving behavior. The fourth hypothesis examines the relationship between intention and behavior, asserting that strong intentions predict consistent implementation of energy-saving practices, though practical barriers may occasionally mediate this relationship. Finally, the fifth hypothesis addresses the role of individual comfort, suggesting that perceived discomfort, particularly in thermal environments, may serve as a trade-off that dampens engagement in energy-saving actions. Together, these hypotheses frame the study's analytical approach, enabling a comprehensive exploration of the factors underpinning energy conservation in culturally nuanced and workplace-specific contexts.

### 3.4 Data collection

This study employed an online survey via Google Forms to gather data from participants working in various private companies in Bangkok, Thailand. The survey link was distributed to company managers, who disseminated it within their organizations. Bangkok was selected as the research site due to its rapid urbanization and prominence as an economic hub, where office buildings significantly contribute to energy consumption. The survey was carried out over 2 months, yielding 105 valid responses. The questionnaire consisted of two sections: the first focused on demographic information, while the second assessed key constructs such as ability, motivation, opportunity, intention, and energy-saving behavior. The survey items were adapted from validated scales in previous studies to ensure consistency and reliability. This data collection method provided a comprehensive dataset for evaluating the proposed modified integrated framework through Partial Least Squares Structural Equation Modeling (PLS-SEM).

### 3.5 Questionnaire design

The questionnaire was divided into two sections. The first section asked for demographic information such as gender, age, education level, and income, and the second section addressed the major measures of this study in the following sequence.

1. Behavioral measures (e.g., turning off electronic devices when they are not in use; see Table 1).
2. Ability (perceived behavioral control; see Table 2).
3. Motivation (personal norms, ascription of responsibility, awareness of consequences, and awareness of need; see Table 2).
4. Opportunity (subjective norms, descriptive norms; see Table 2).
5. Intention (Table 2).
6. Individual comfort (Table 2).

**Table 1:** Survey item.

**Constructs**	**Description**
Energy-saving behaviors	How often do you turn off the following devices when they are not in use for saving energy? (1) Light; (2) Computer; (3) Air conditioner (AC).

**Table 2:** Measurement items for constructs.

**Constructs**	**Item no**.	**Items**
Intention	I1	1. I will try to save energy in my workplace.
	I2	2. I am willing to save energy in my workplace.
Individual comfort	IC1	1. I am satisfied with the temperature in my workplace.
	IC2	2. I am satisfied with the light in my workplace.
	IC3	3. I am satisfied with the indoor environment of my workplace.
**Ability**		
1. Perceived behavioral control	PCB1	1. Increasing energy-saving behavior in the workplace is entirely within my control.
	PCB2	2. I am confident that I can save energy in the workplace if I want to.
	PCB3	3. I have the knowledge and skills to save energy in the workplace.
**Motivation**		
1. Awareness of consequences	AC1	1. When I reduce electricity use in my workplace, I am doing something good.
	AC2	2. When I reduce electricity use in my workplace, I am reducing costs.
2. Ascription of responsibility	AR1	1. I feel responsible for the energy use in the workplace.
	AR2	2. I feel responsible for reducing energy use in the workplace.
3.Personal norm	PN1	1. I feel good about myself when I do not use a lot of energy.
	PN2	2. I feel guilty when I use a lot of energy in the workplace.
	PN3	3. I think I have a responsibility to save energy in the workplace.
4. Awareness of Need	AN1	1. Energy saving in my workplace can contribute the sustainability of our society.
	AN2	2. Energy saving in my workplace contributes to alleviating energy shortage issues.
**Opportunity**		
1. Subjective norms	S1	1. Most of my colleagues expect me to turn off the computer when leaving.
	S2	2. Most of my colleagues expect me to turn off the light when leaving.
2. Descriptive norms	D1	1. The management team has taken actions to save energy.

The measures were taken from previous studies (Carrico and Riemer, 2011; Chen and Knight, 2014; Li et al., 2019; Xu et al., 2020). The variables were evaluated on a five-point Likert scale, with a minimum of one and a maximum of five. Table 2 shows the integrated model's survey structure and detailed measures.

### 3.6 Data analysis method

The data were analyzed using Partial Least Squares Structural Equation Modeling (PLS-SEM), a method well-suited for testing complex models and small sample sizes. SmartPLS 3.0 software was used to perform the analysis, which followed a two-step approach (Wong, 2013). First, the measurement model was evaluated to ensure the reliability and validity of the constructs. Reliability was assessed using Cronbach's alpha and composite reliability, while convergent validity was examined through the average variance extracted. Discriminant validity was tested using the Heterotrait-Monotrait (HTMT) ratio to ensure that constructs were distinct. The second step evaluated the structural model to test the hypothesized relationships between constructs. Path coefficients, effect sizes, and the coefficient of determination (*R*^2^) were calculated to assess the strength and significance of these relationships. Bootstrapping with 5,000 resamples was conducted to ensure statistical robustness.

## 4 Analysis of data and results

The PLS-SEM analysis was conducted using SmartPLS, evaluating both the measurement and structural models. The measurement model assessed the validity and reliability of latent constructs, while the structural model examined the relationships between independent and dependent variables (MacCallum and Browne, 1993). The measurement model was assessed to ensure the reliability and validity of the constructs before proceeding with the structural model analysis. All constructs met the required thresholds for composite reliability (CR), Cronbach's alpha, average variance extracted (AVE), and discriminant validity, confirming the robustness of the measurement model. The structural equation modeling (PLS-SEM) analysis provided statistical support for all hypothesized relationships; however, three constructs—opportunity, intention, and individual comfort—exhibited negative directional effects on energy-saving behavior.

### 4.1 Descriptive statistics

The study surveyed 105 office workers in various private-sector organizations across Thailand, capturing a diverse demographic representative of modern Thai office environments. The gender distribution was balanced, with 50.5% male and 49.5% female respondents. Age groups were well-distributed, with the majority (38.1%) aged between 35 and 45 years, followed by 31.5% aged 25–35. Educational attainment was predominantly at the bachelor's level (66.6%), with 18.1% holding master's degrees. These figures align with the profile of urban professionals in Thailand's private sector. Monthly income levels ranged primarily from 15,000 to 30,000 Baht (56.2%), a demographic reflective of middle-class office workers. Participants' professional roles included mid-level administrators (45%), managers (35%), and technical staff (20%), ensuring a robust representation of organizational hierarchies and responsibilities. This demographic distribution provides a strong contextual foundation for examining the interplay of individual, organizational, and cultural influences on energy-saving behaviors in Thai office environments. Survey findings reveal that most participants demonstrated a strong commitment to energy-saving practices, as reflected in a mean intention score of 4.2 on a 5-point scale. Approximately 85% of respondents reported high levels of awareness about energy conservation, scoring 4 or above, and 72% acknowledged a personal sense of responsibility for contributing to energy-saving actions. The construct of ability, which represents perceived behavioral control and confidence in implementing conservation measures, achieved a mean score of 3.9, indicating moderate confidence among participants. Opportunity, measuring external support and organizational enablers, received a lower mean score of 3.5, with 55% of respondents agreeing that their workplace provided sufficient resources and policies to support energy-saving behaviors, indicating potential structural and organizational support gaps.

### 4.2 Model validation

#### 4.2.1 Reliability and convergent validity

Ensuring the reliability and validity of constructs is fundamental to the robustness of the theoretical framework. Reliability was assessed using Cronbach's alpha and composite reliability (CR), which evaluates the internal consistency of measurement scales. Cronbach's alpha values exceeding the 0.70 threshold indicate that the items within each construct reliably measure the intended dimension. Composite reliability further validates this by accounting for shared variance among indicators. Convergent validity was evaluated using average variance extracted (AVE), with a threshold of 0.50, indicating that the construct explains a substantial portion of variance. Table 3 provides the reliability and validity results, confirming that all constructs exceeded the recommended thresholds. For instance, motivation achieved a Cronbach's alpha of 0.913, a CR of 0.930, and an AVE of 0.628, demonstrating its strong reliability and convergent validity. Similarly, constructs such as Personal Norms and Ascription of Responsibility also performed well, with AVE values of 0.695 and 0.696, respectively. These results confirm that the constructs are robust and aligned with the theoretical framework.

**Table 3:** Reliability and validity.

	**Cronbach's alpha**	**Composite reliability**	**Average variance extracted (AVE)**
Ability	0.761	0.768	0.542
Ascription of responsibility	0.763	0.821	0.696
Awareness of consequence	0.744	0.847	0.735
Behavior	0.79	0.821	0.635
Motivation	0.913	0.93	0.628
Opportunity	0.747	0.757	0.517
Personal norm	0.853	0.901	0.695

#### 4.2.2 Discriminant validity

Discriminant validity ensures that constructs are distinct and measure unique theoretical dimensions. The Heterotrait-Monotrait (HTMT) ratio was used to assess discriminant validity, with values below 0.85 confirming that constructs are conceptually separate. Table 4 presents the HTMT ratios, indicating satisfactory discriminant validity for all constructs. For example, the HTMT value between Motivation and Intention was 0.794, confirming a strong yet distinct relationship. These findings underscore the theoretical clarity and conceptual robustness of the model.

**Table 4:** Discriminant validity (HTMT).

	**Ability**	**Energy-saving behavior**	**Individual comfort**	**Intention**	**Motivation**	**Opportunity**
**Ability**	–	–	–	–	–	–
**Energy-saving behavior**	0.804	–	–	–	–	–
**Individual comfort**	0.848	0.850	–	–	–	–
**Intention**	0.791	0.321	0.764	–	–	–
Motivation	0.766	0.471	0.779	0.794	–	–
Opportunity	0.617	0.730	0.691	0.609	0.425	–

### 4.3 Path analysis

**Figure 3:**
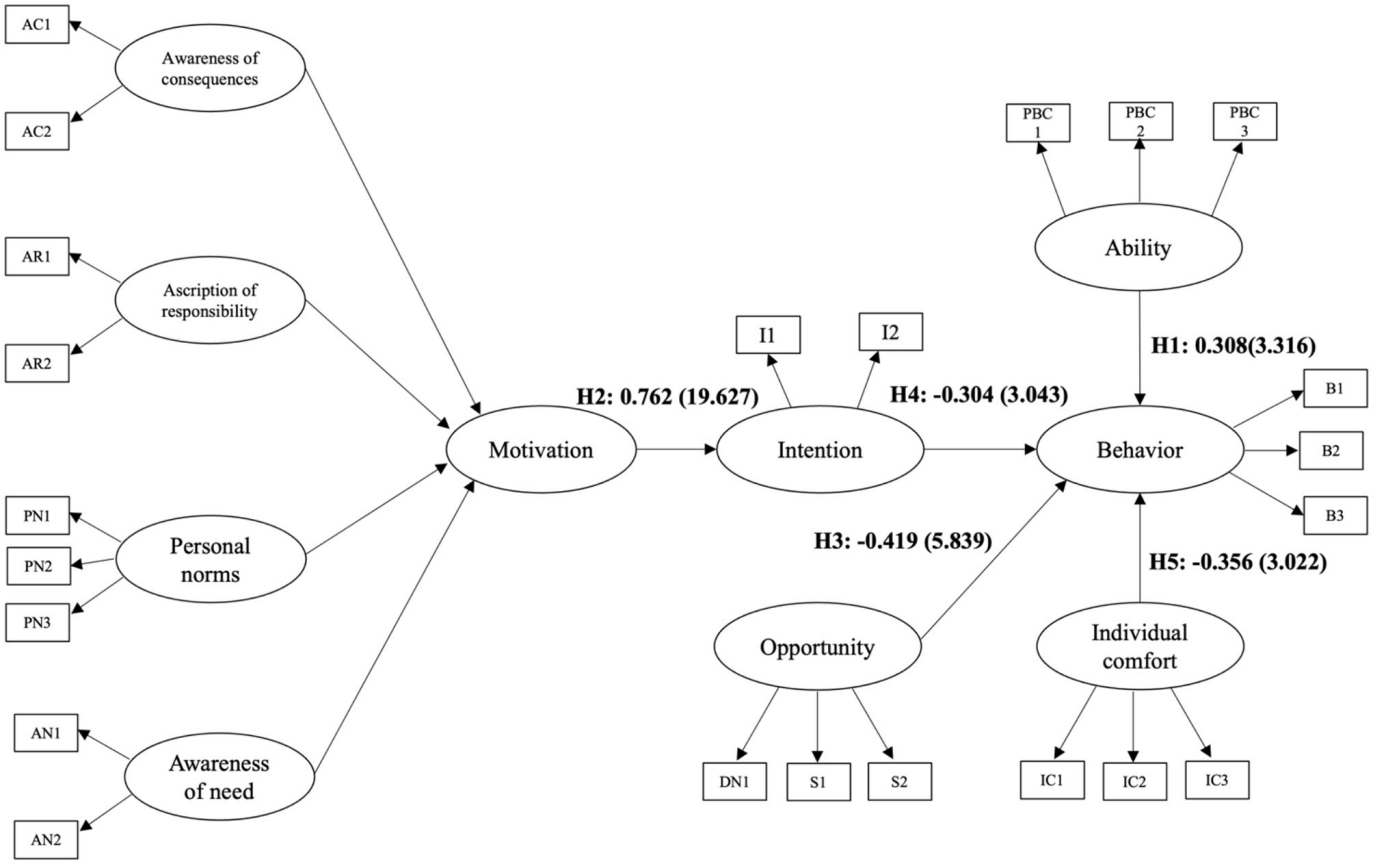
Results of the modified integrated model.

The hypothesized relationships were all statistically significant (*p* < 0.01), confirming their relevance within the model. However, three relationships, opportunity, intention, and individual comfort with energy-saving behavior, demonstrated negative effects, diverging from the conventional directional expectations. These relationships are depicted in Figure 3, which provides a visual representation of the validated pathways among the constructs. Additionally, Table 5 presents the effect sizes (*f*^2^) associated with each relationship, offering further insight into the practical significance of the findings. The pathway from ability to energy-saving behavior was positive and statistically significant (β = 0.308, *p* < 0.001), with a large effect size (*f*^2^ = 1.02). This result underscores the pivotal role of perceived control in facilitating pro-environmental behaviors. Similarly, the motivation to intention relationship was strongly positive (β = 0.762, *p* < 0.001), accompanied by a medium-to-large effect size (*f*^2^ = 0.582). These findings align with existing literature on the influence of personal norms and awareness of consequences in shaping sustainability-related intentions in the workplace. In contrast, the opportunity to energy-saving behavior relationship exhibited an unexpected negative effect (β = −0.419, *p* < 0.001), with an effect size of *f*^2^ = 0.33. This suggests that contextual factors, such as workplace policies or social norms, may be perceived as constraints rather than enablers of energy-saving behaviors. The intention to energy-saving behavior relationship also demonstrated a negative effect (β = −0.304, *p* < 0.01), with an effect size of *f*^2^ = 0.24. This points to the presence of an intention-behavior gap, where stated intentions do not always translate into actual behavior, potentially due to competing priorities or external constraints. Finally, the individual comfort to energy-saving behavior relationship was negative (β = −0.356, *p* < 0.01), with an effect size of *f*^2^ = 0.31. This indicates that office workers' prioritization of thermal and visual comfort may hinder their engagement in energy-saving practices, in line with adaptive thermal comfort models that highlight the trade-off between energy efficiency and perceived well-being. The results presented in Figure 3, together with the effect sizes detailed in Table 5, provide strong empirical support for the integrated framework. These findings offer valuable insights into the complex interplay of motivational, contextual, and personal factors in shaping energy conservation efforts within Thai office environments.

**Table 5:** Effect size (*f*^2^).

	**Ability**	**Energy-saving behavior**	**Individual's comfort**	**Intention**	**Motivation**	**Opportunity**
**Ability**		1.02				
**Energy-saving behavior**						
**Individual's comfort**		0.226				
**Intention**		0.46		0.582		
**Motivation**						
**Opportunity**		0.444				

### 4.4 Hypothesis testing

The hypothesis testing results, summarized in Table 6, provide empirical validation for the framework. Although all hypotheses were statistically supported, the negative direction of H3, H4, and H5 challenges conventional behavioral models. These findings indicate that opportunity, intention, and comfort do not always function as enablers of energy conservation but can, under certain conditions, act as constraints.

**Table 6:** Results of path analysis and hypotheses test.

	**Path coefficients**	**STDEV**	***T*-statistics**	***P*-values**	**Support**
**Ability** **>** **Energy-saving behavior**	0.308	0.093	3.316	0.001	Yes
**Motivation** **>** **Intention**	0.762	0.039	19.627	0.000	Yes
**Opportunity** **>** **Energy-saving behavior**	−0.419	0.072	5.839	0.000	Yes
**Intention** **>** **Energy-saving behavior**	−0.304	0.100	3.043	0.002	Yes
**Individual's comfort** **>** **Energy-saving behavior**	−0.356	0.118	3.022	0.003	Yes

## 5 Discussion and implications

### 5.1 Interpreting the unexpected negative effects

The findings of this study reveal several unexpected relationships, particularly the negative effects of opportunity, intention, and individual comfort on energy-saving behavior. First, the negative effect of opportunity on energy-saving behavior suggests that workplace structures and organizational norms may not always facilitate conservation actions. Previous studies have generally assumed that the presence of energy-saving policies and workplace support leads to higher engagement in sustainable practices. However, our findings align with behavioral resistance theories, which suggest that when individuals perceive environmental policies as externally imposed rather than self-directed, they may actively disengage from the desired behavior. Office workers might view certain conservation initiatives as inconvenient or misaligned with their work habits, leading to psychological reactance, where individuals resist changes that limit their autonomy. Second, the negative relationship between intention and energy-saving behavior highlights the well-documented intention-behavior gap, wherein stated intentions do not always translate into actual actions. Several possible explanations exist for this discrepancy. Office workers may express willingness to save energy, but situational constraints such as high workload demands, ingrained workplace habits, or a lack of direct incentives could prevent them from acting accordingly. Moreover, workplace energy-saving behaviors often rely on social reinforcement, meaning that without visible peer engagement or direct accountability, office workers may deprioritize conservation efforts despite their stated intentions. Finally, the negative association between individual comfort and energy-saving behavior suggests that Office workers prioritize thermal and lighting comfort over sustainability. Unlike household energy-saving decisions, where individuals directly benefit from lower energy costs, workplace environments often detach personal benefits from conservation behaviors. Office workers may feel that reducing air conditioning or adjusting lighting levels has little direct impact on their well-being but could compromise their comfort, productivity, or job satisfaction. This aligns with findings from adaptive thermal comfort models, which emphasize that personal comfort often overrides sustainability concerns in decision-making.

### 5.2 Theoretical contributions

This study advances the literature on workplace energy conservation by providing new insights into the complex relationships among motivation, opportunity, ability, intention, and individual comfort in shaping energy-saving behavior. While previous research often assumes that opportunity, intention, and comfort facilitate conservation efforts, the findings suggest that their effects are more nuanced and context-dependent. In contrast, the results confirm the positive roles of ability and motivation, reinforcing the importance of perceived behavioral control and intrinsic drivers in sustainability models. The negative effect of opportunity on energy-saving behavior challenges a key assumption of the Motivation-Opportunity-Ability (MOA) model, which traditionally views opportunity as an enabler. These findings indicate that when workplace energy-saving policies are perceived as externally imposed rather than empowering, they may induce psychological resistance, reducing engagement. This perspective aligns with behavioral resistance theories, which emphasize that rigid policies can sometimes act as constraints rather than facilitators. Such insights refine the understanding of how structural and social influences shape workplace sustainability behavior. Another key finding highlights the disconnect between intention and behavior, reinforcing the intention-behavior gap discussed in the Theory of Planned Behavior (TPB). Although TPB assumes that strong intentions predict behavior, the results suggest that external workplace constraints, such as workload pressures or the absence of visible reinforcement, can weaken this relationship. This challenges the assumption that intention alone is a sufficient predictor of energy conservation, particularly in structured organizational environments. Moreover, the study contributes to adaptive thermal comfort research by demonstrating that individual comfort preferences may sometimes conflict with sustainability efforts. Unlike prior models that assume individuals adjust their behaviors to environmental conditions, the results indicate that office workers may prioritize personal comfort over energy efficiency, particularly when energy-saving measures require significant trade-offs. This perspective refines current energy-saving behavior models by incorporating the role of workplace comfort as a potential barrier to conservation efforts.

Despite these unexpected negative effects, the findings confirm the strong positive impact of ability and motivation on energy-saving behavior. Office workers who possess a greater sense of control and confidence in their ability to conserve energy are more likely to engage in sustainable actions. Likewise, individuals with higher intrinsic motivation, driven by personal norms, moral responsibility, and awareness of consequences, demonstrate stronger energy-saving intentions. These findings reinforce the role of perceived behavioral control within TPB and highlight the critical role of intrinsic motivation in pro-environmental workplace behaviors. By integrating MOA, NAM, and TPB, this study extends theoretical models by demonstrating how psychological, organizational, and contextual factors interact to shape workplace energy-saving behaviors. Furthermore, the results challenge overly simplistic assumptions regarding opportunity, intention, and comfort, offering a more nuanced understanding of workplace energy conservation. These insights contribute to the refinement of behavioral theories and support the development of more context-sensitive models for understanding sustainability in organizational settings.

### 5.3 Practical implications

The findings of this study offer actionable insights for promoting energy-saving behaviors in workplace environments, particularly within the Thai context. These implications address organizational practices and broader policy initiatives, ensuring that interventions align with cultural and environmental factors. One key practical implication is the need to foster energy-saving cultures within organizations. This study demonstrates that moral responsibility and collective accountability are pivotal in shaping office workers' behaviors. Programs that leverage peer influence and management-led initiatives can align individual actions with organizational sustainability goals. For example, Su et al. (2022) found that cultivating an energy-conscious workplace culture enhances behavioral engagement, a finding supported by the strong role of motivation in this study. The research also underscores the importance of addressing structural barriers to energy-saving behaviors. The negative relationship between opportunity and energy-saving behaviors highlights challenges such as inadequate resources, unclear policies, and lack of organizational support. Providing accessible energy-efficient technologies, clear operational guidelines, and training programs can mitigate these barriers, enabling workers to adopt sustainable practices more effectively (Chen and Chen, 2021). Balancing thermal comfort and conservation goals emerges as another critical area for intervention. Findings from this study indicate that individual comfort significantly influences energy-saving behaviors, suggesting that rigid energy-saving measures may face resistance. Flexible and adaptive thermal control systems and office workers' feedback mechanisms can help organizations balance comfort and conservation without undermining productivity (Iamtrakul et al., 2020). Finally, regional considerations specific to Thailand, such as the prevalence of urban heat islands and the increasing adoption of hybrid work models, should inform energy-saving strategies. Tailored interventions that address these contextual factors, such as optimizing energy use during peak hours or integrating hybrid-friendly policies, can enhance the effectiveness of workplace sustainability initiatives (Mantesi et al., 2022; Hnin et al., 2024). These practical recommendations provide a roadmap for organizations and policymakers to design effective energy-saving interventions that are both culturally relevant and operationally feasible. By addressing the interplay of motivation, opportunity, and ability within specific workplace contexts, this research offers a comprehensive approach to achieving sustainable energy management.

### 5.4 Limitations and future research

While this study provides important insights, several limitations should be acknowledged. The sample size (*n* = 105) limits the generalizability of the findings, particularly regarding the unexpected negative relationships. Future research should employ larger, more diverse samples across different industries and cultural contexts to validate these results. Moreover, the study's cross-sectional design does not capture long-term behavioral changes. Longitudinal studies are needed to examine whether workplace energy-saving behaviors evolve, particularly as organizational cultures shift and policies adapt. While this study focuses on behavioral predictors, future research could explore the role of emerging technologies, such as AI-driven energy management systems, workplace automation, and digital nudges, in shaping conservation behaviors. Integrating behavioral insights with smart technology solutions could provide more effective, scalable interventions for workplace sustainability. By addressing these limitations, future research can build on the insights provided by this study, refining the framework for broader applicability and impact.

## 6 Conclusion

This study contributes to the growing body of research on workplace energy conservation by examining how motivation, opportunity, ability, intention, and individual comfort influence energy-saving behavior. Using an integrated framework that combines the Motivation-Opportunity-Ability (MOA) model, the Norm Activation Model (NAM), and the Theory of Planned Behavior (TPB), the study provides insights into the complex factors shaping energy-saving behaviors in office environments in Thailand. The findings confirm that motivation and ability positively influence energy-saving behavior, highlighting the importance of intrinsic drivers (personal norms and awareness of consequences) and perceived control in shaping conservation actions. However, opportunity, intention, and individual comfort exhibited unexpected negative relationships with energy-saving behavior. Rather than dismissing these results as purely statistical anomalies, this study suggests that organizational constraints, psychological resistance, and thermal comfort trade-offs may explain why workplace conservation efforts do not always lead to expected behavioral changes. From a theoretical perspective, these findings challenge simplistic assumptions in behavioral models that assume external enablers (opportunity) and self-reported intentions always lead to pro-environmental actions. Instead, the study highlights the need to consider policy perception effects, social norm reinforcement, and behavioral inertia in understanding workplace energy-saving behaviors. The integration of adaptive comfort models and behavioral resistance theories provides a more nuanced framework for future sustainability research. From a practical standpoint, the study underscores the need for organizations to rethink energy-saving interventions. Effective policies should not only provide resources and structural support but also address office workers' autonomy, real-time behavioral feedback, and workplace comfort needs. The study suggests that participatory policy design, digital energy monitoring tools, and incentive-driven conservation programs could help bridge the gap between intention and action. Despite its contributions, this study has several limitations. The sample size (*n* = 105), while appropriate for PLS-SEM analysis, remains relatively small, which may have amplified suppressor effects. Future research should validate these findings with larger, cross-industry samples to improve generalizability. Additionally, this study employed a cross-sectional design, limiting insights into long-term behavioral shifts. Longitudinal research could assess how energy-saving behaviors evolve over time and whether policy adjustments lead to sustained engagement. Another avenue for future research is exploring the role of emerging technologies in workplace energy conservation. With advancements in AI-driven energy management systems, behavioral nudging via smart office solutions, and gamification-based sustainability programs, future studies could examine how digital interventions influence conservation behavior.

## Data Availability

The original contributions presented in the study are included in the article/supplementary material, further inquiries can be directed to the corresponding author.
